# Changes in emotional dysregulation profile among offspring of parents with bipolar disorder: a family-based intervention pilot study

**DOI:** 10.1186/s40345-025-00398-3

**Published:** 2025-11-03

**Authors:** Erica Fongaro, Florence Pupier, Marie-Christine Picot, Nathalie Franc, Marion Soler, Emilie Olié, Virginie Maurice, Diane Purper-Ouakil, Hala Kerbage

**Affiliations:** 1 Centre Hospitalier Universitaire de Montpellier, Saint Eloi Hospital, Montpellier, Hérault, France; 2 CESP INSERM U 1018 UVSQ Psychiatry Development and Trajectories, Villejuif, France; 3 Clinical & Epidemiological Research Unit, CHU, Montpellier, Montpellier, France; 4 Department of Emergency Psychiatry and Acute Care, Lapeyronie Hospital, CHU Montpellier, Montpellier, France; 5 IGF, Univ. Montpellier, CNRS, INSERM, Montpellier, France

**Keywords:** Bipolar disorder, Offspring, CBCL, Emotional dysregulation, Family-focused treatment

## Abstract

**Abstract:**

Offspring of parents with bipolar disorder (OBD) are at increased risk for mood and behavioral disorders, with emotional dysregulation being an early marker. This pilot study aimed to evaluate the effect of a family-focused intervention on emotional dysregulation in OBD, hypothesizing a significant reduction in CBCL Emotional Dysregulation Profile scores over 12 months. Twenty-five OBD aged 6–16 years participated in a four-session psychoeducational program. Significant reductions were observed in CBCL emotional dysregulation scores suggesting that a brief family-focused program may reduce dysregulation and improve functioning in OBD.

**Trial registration number:**

Clinicaltrials.gov. ID NCT03299140. Registered on the 17th September, 2017.

## Introduction

Bipolar disorder (BD) is a chronic mood disorder with early onset and high heritability (70–80%) [1], affecting 1–2% of adults [2]. Parents with BD often exhibit mood instability and impaired caregiving, contributing to disrupted parent–child relationships [3–5]. Their offspring (OBD) are at significantly elevated risk for mood, anxiety, behavioural, and attentional disorders [5].

Emotional dysregulation—a transdiagnostic marker of severe psychopathology—is common in OBD and may precede formal diagnosis [6]. The Child Behavior Checklist Emotional Dysregulation Profiles (CBCL-EDP), reflecting attention, aggression, and anxiety/depression, is a validated early indicator of emotional and behavioral instability [7].

Despite well-documented vulnerability, preventive interventions for asymptomatic but high-risk youth remain scarce [8]. Most programs target al.ready symptomatic children, leaving many at familial risk unsupported [9]. Recent trials highlight the promise of family-based preventive interventions [10]. Family-Focused Treatment (FFT) is one such program shown to reduce conflict and improve emotion regulation in at-risk youth [11].

Drawing on FFT principles, we developed a brief family-based intervention within a Child and Adolescent Mental Health Service (CAMHS) in France. This pilot study evaluated its impact on emotional dysregulation in OBD, hypothesizing a significant reduction in CBCL emotional dysregulation profile scores over 12 months.

## Materials and methods

### Study design and participant recruitment

This monocentric observational pilot study recruited OBD aged 6–16 years and at least one parent with BD from the Bipolar Expert Center (CHU Montpellier) and community referrals over a 24-month period (2014–2016). Of the 49 families initially contacted, 42 met inclusion criteria, and 25 enrolled and completed the intervention, representing an attrition rate of 40.5%. Inclusion required informed consent and parental clinical stability based on their clinician’s judgement. Offspring psychiatric symptoms were not required for inclusion; all children of parents with bipolar disorder aged 6–16 years were eligible, regardless of clinical status. This approach allowed both symptomatic and asymptomatic youth at familial risk to participate, reflecting the program’s preventive aims. Among symptomatic participants, many had chronic or recurrent conditions such as ADHD or anxiety disorders, but illness duration was not a selection criterion. Exclusion criteria were a current mood episode, ASD, intellectual disability, or any condition hindering group participation, in either parent or child. This study was approved by the ethics committee “COMITE DE PROTECTION DES PERSONNES SUD-MEDITARRANEE I” (IRB ID: 2014-A00204-43). All participants provided written informed consent prior to their inclusion.

## Intervention

The intervention consisted of a family-based program adapted from Miklowitz’s FFT, a cognitive-behavioral approach combining psychoeducation, communication enhancement, and problem-solving training originally developed for early-onset BD. Our adaptation focused on families where only the parent had BD, aiming to strengthen offspring coping skills, family communication, and early responses to emotional dysregulation. Four 3-hour sessions were delivered at the St Eloi CAMHS over six months, approximately every six weeks, by experienced clinicians trained in family and cognitive-behavioural therapy Sessions were intentionally spaced to allow families time to practice skills at home between sessions. Two parallel groups ran simultaneously:

Parents ‘group: one session on psychoeducation about BD symptoms, aetiology, heritability, and offspring vulnerability; two sessions on educational strategies for reducing familial conflict, establishing stable routines (e.g., sleep hygiene), and managing emotional dysregulation using behavioural strategies (e.g., time-out, “special time” routines inspired by Barkley’s program); and one session on communication skills for labelling emotions, encouraging positive behaviours, and fostering family cohesion.

Offspring group: one session on psychoeducation about parental BD with open discussion; one session on emotion regulation strategies (e.g., anger management techniques); one session on communication skills (e.g., active listening, assertiveness); and one session on problem-solving and stress management.

Unlike standard FFT protocols involving 12–21 conjoint sessions, our adaptation used four sessions combining separate parent and youth groups with brief joint activities and structured homework assignments. This format allowed age-appropriate psychoeducation and skills training while reducing attendance barriers common in routine care. Parents and youth practiced communication and problem-solving techniques together at home using guided exercises, ensuring that key FFT components were retained despite fewer in-person conjoint sessions. Table 1 summarizes the content of each session.

**Table 1:** Overview of the family-focused program for emotional dysregulation in OBD: content themes and activities

Session	Parents’ group content	Offspring group content	Format & activities
**Session 1**	Psychoeducation: symptoms, etiology, heritability of BD, offspring vulnerability	Psychoeducation about parental BD, open discussion on questions/concerns	Interactive presentations, Q&A, group discussions
**Session 2**	Educational strategies: reducing familial conflict, establishing routines (e.g., sleep hygiene), understanding emotional dysregulation, prevention strategies	Emotion regulation strategies: identifying emotions, anger management tools	Role-playing, small-group exercises, skills practice
**Session 3**	Communication skills: labeling emotions, praising positive behaviors, family communication strategies	Communication skills: active listening, assertiveness training, expressing emotions safely	Communication games, modeling by facilitators, family-based exercises
**Session 4**	Problem-solving approaches: stress management, early detection of relapse, collaborative problem-solving	Problem-solving and coping strategies: stress management, confidence-building	Joint activities with parents and youth, guided problem-solving practice, goal-setting exercises

## Measures

Participants were assessed at baseline (T0), 6 months (T1), and 12 months (T2) using a standardized battery of validated instruments. Emotional and behavioral problems were evaluated primarily using the CBCL 4–18 [12, 13], with the EDP score, serving as the main outcome measure. The CBCL-EDP were calculated as the summed T-scores of the Anxious/Depressed, Attention Problems, and Aggressive Behavior subscales. The Kiddie-Schedule for Affective Disorders and Schizophrenia – Present and Lifetime Version (Kiddie-SADS-PL) [14] was used to establish DSM-based psychiatric diagnoses and conducted with both the child and at least one parent. Dimensional ratings of emotional and behavioural adjustment were obtained via the Strengths and Difficulties Questionnaire (SDQ) [15] and was completed by parents and by youth aged ≥ 11 years (self-report version). Functional impairment was assessed using the Children’s Global Assessment Scale (CGAS) [16]. Mood symptoms were evaluated using the Children’s Depression Rating Scale – Revised (CDRS-R) [17] for depressive symptoms. Attention and hyperactivity were assessed with the ADHD Rating Scale IV (ADHD-RS-IV) [18]. The Wechsler Abbreviated Scale of Intelligence (WASI) was administered at baseline (T0) by trained clinicians to assess youths’ general intellectual functioning [19]. All other measures were administered at T0, T1, and T2, except for the ADHD-RS-IV, which was administered only at T0 and T2.

## Statistical analyses

CBCL-EDP changes and other outcome measures were evaluated using paired t-tests or Wilcoxon signed-rank tests. Correlations used Spearman *ρ* and Pearson *r*. Analyses were conducted in SAS v9; significance was *p* <.05.

## Results

### Participants and clinical characteristics

The clinical and demographic characteristics are presented in Table 2 for children and Table 3 for parents. Of the 49 families contacted, 42 met eligibility criteria, and 25 enrolled in the study, corresponding to a participation rate of 59.5%. The attrition rate of 40.5% reflects eligible families who declined participation prior to enrolment. All 25 enrolled families completed the full program (i.e., all four sessions), and no families dropped out once the intervention had begun. 84.0% of participants met criteria for at least one psychiatric diagnosis based on the KSADS-PL, 43.0% met criteria for more than one diagnosis, and 16.0% did not meet criteria for any psychiatric diagnosis. At baseline, 60% of youth scored below 70 on the CGAS, reflecting significant functional impairment. Regarding emotional dysregulation, 40% were in the Deficient Emotional Self-Regulation (DESR) range and 28% met criteria for the Dysregulation Profile (DP ≥ 210) (Table 2).

**Table 2:** Description of OBD clinical and demographic characteristics

Variable	Category	*N* = 25
Gender	Girls	14 (56.0%)
	Boys	11 (44.0%)
Age (years)	Mean (± DS)	10.87 (2.34)
School level	Elementary and middle school	14 (56.0%)
	High School	11 (44.0%)
Parental authority	Both parents	19 (76.0%)
Living with both bio-parents	Both biological parents (married or shared care)	22
	Bipolar parent only	3
Current treatment	Medication	7 (28.0%)
	Day unit hospitalization	3 (12.0%)
CGAS (T0)	Mean (± DS)	62.92 (16.48)
	≥ Cut-off = 70	10 (40.0%)
	< Cut-off = 70	15 (60.0%)
CBCL (T0)	No Emotional Dysregulation	8 (32.0%)
	Deficient Emotional Self-Regulation	10 (40.0%)
	Dysregulation Profile	7 (28.0%)
KSADS	ADHD	16 (64.0%)
	Oppositional Defiant Disorder	6 (24.0%)
	Separation Anxiety Disorder	9 (36.0%)
	Major Depressive Disorder	8 (32.0%)
	Generalized Anxiety Disorder	8 (32.0%)
	Sleeping disorder	6 (24.0%)
	Mania	2 (8.0%)
	Post‑Traumatic Stress Disorder	4 (16.0%)

1) In the French school system, élémentaire (ages 6–10) and collège (ages 11–14) were combined as “Elementary/Middle school” to facilitate comparison with international terminology. High school refers to lycée (ages 15–18)

2) “Parental authority” refers to legal custody and medical decision-making rights whereas “Living with both biological parents” refers to the child’s primary residence

3) Treatment categories are not mutually exclusive; some youth received more than one type of intervention. Psychiatric/psychological care includes outpatient psychiatric or psychological treatment, whereas psychoeducation refers to structured, group- or family-based educational programs about mental health conditions and coping strategies

4) DESR (Deficient Emotional Self-Regulation) and DP (Dysregulation Profile) are based on the summed T-scores of the Attention Problems, Aggressive Behavior, and Anxious/Depressed subscales. DESR = 180–210; DP ≥ 210

5) For the KSADS-PL, Percentages indicate the proportion of participants meeting DSM-IV criteria for each diagnosis based on the KSADS-PL. Categories are not mutually exclusive

**Table 3:** Description of BD parents’ clinical and demographic characteristics

Variable	Category	n (%) *N* = 25
Gender	Women	15 (60.0%)
	Men	10 (40.0%)
Age (years)	Mean	43,16 (± 5,34)
BD Type I		14 (56.0%)
BD Type II		6 (24.0%)
BD Type III		2 (8.0%)
BD Not Specified		3 (12.0%)
Current Treatment	Medication (mood stabilizer)	7 (28.0%)
	No mood stabilizer	7 (28.0%)
	Current inpatient hospitalization	3 (12.0%)

Treatment categories are not mutually exclusive; parents may have received more than one type of treatment. Current hospitalization refers to parents who were still formally hospitalized at baseline but were clinically stable and in the final phase of their inpatient stay, thus meeting inclusion criteria. None were in the acute phase at study entry. Medication (mood stabilizer) refers to parents prescribed at least one mood stabilizer; no mood stabilizer refers to those receiving only other psychotropic classes (e.g., antidepressants, antipsychotics)

## Primary and secondary outcomes

**Fig. 1:**
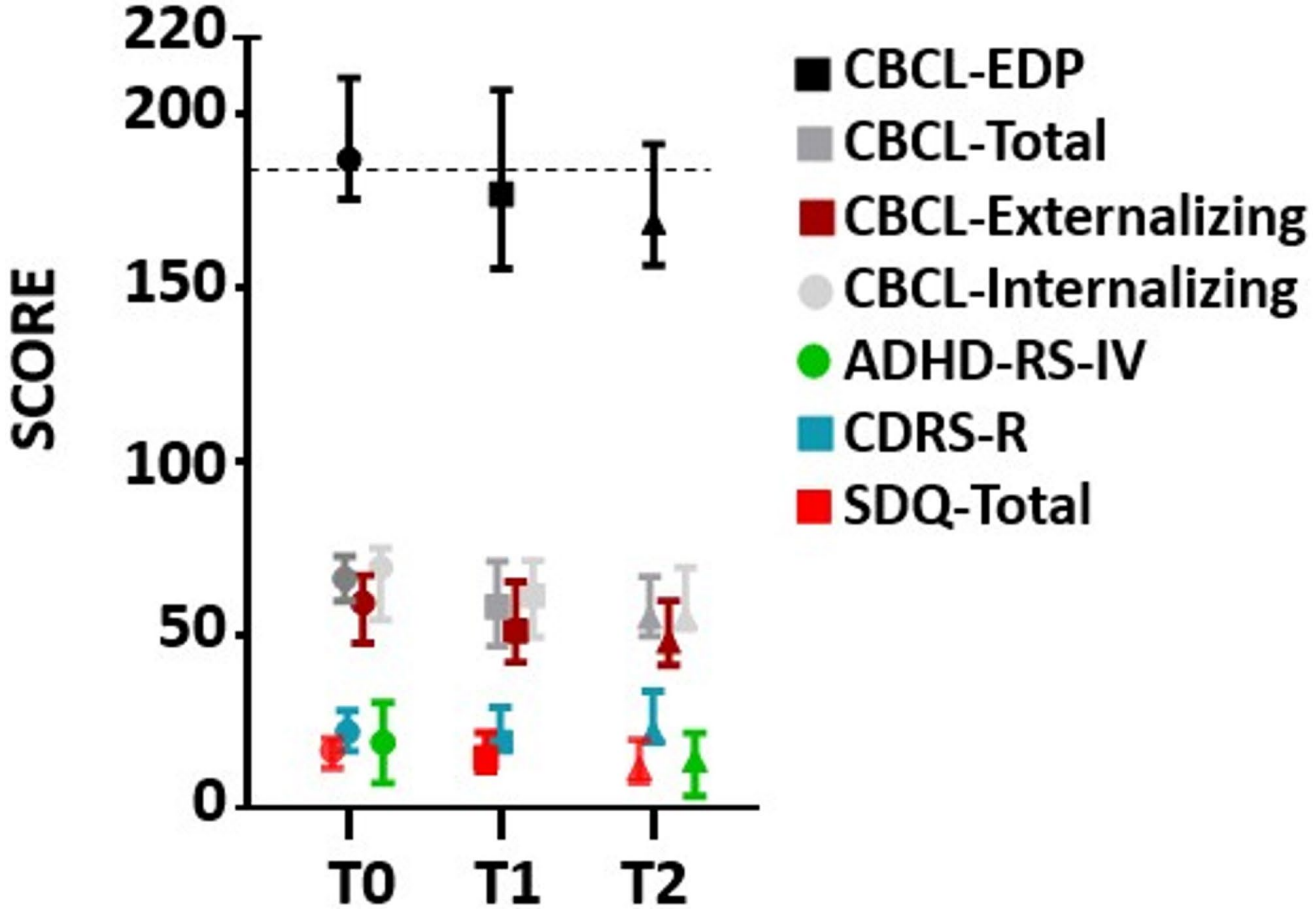
Measures mean scores at baseline, 6 months and 12 months

CBCL-EDP did not differ significantly from T0 (M = 191.76) to T1 (M = 182.9), t(44) = 1.12, *p* =.27. and significantly declined from T0 to T2 (M = 176.88; mean difference = −14.88; t(24) = −4.48; *p* <.001). (Fig. 1). This change was unrelated to gender, age, education, parental BD type or prior treatment and family structure. There were non-significant improvements across the total score of other outcome measures from T0 to T1 (CBCL-Total Score: t(44) = 1.45, *p* =.15; CBCL Externalizing: t(44) = 0.99, *p* =.32; CBCL Internalizing: t(44) = 1.46, *p* =.15; CDRS-R: Mann–Whitney U = 231.5, *p* =.89; SDQ: t(42) = 0.25, *p* =.80; CGAS: t(44) = 1.45, *p* =.15). The CBCL-Total Score Score significantly improved from T0 to T2 (CBCL-Total Score: t(48) = 2.00, *p* =.05). There were no significant improvements across the other outcomes (CBCL Externalizing: t(48) = 1.85, *p* =.07; CBCL Internalizing: t(48) = 1.92, *p* =.06; CDRS-R: Mann–Whitney U = 233, *p* =.37; SDQ: t(46) = 1.32, *p* =.19; CGAS: t(44) = 1.45, *p* =.15; ADHD-RS-IV: t(45) = 1.04, *p* =.30) (Fig. 1). Group comparisons were conducted between participants with and without a psychiatric diagnosis to evaluate differences in CBCL-EDP score changes (Table 4).

**Table 4:** Group comparisons between participants with and without a psychiatric diagnosis and the CBCL-EDP score changes

Baseline measure (KSADS)	With	Without	*p*-value
ADHD	−7.50 (± 9.24)	−19.80 (± 18.78)	0.04*
Anxiety Disorders	−7.22 (± 9.23)	−19.19 (± 18.45)	0.08
Oppositional Defiant Disorder	−15.42 (± 17.80)	−10.20 (± 12.52)	0.55
Sleep Disorders	−15.39 (± 15.90)	−19.17 (± 14.05)	0.48
Depression	−16.36 (± 15.17)	−4.00 (± 26.23)	0.23

## Discussion

This pilot study assessed the effects of a brief family-based intervention targeting emotion dysregulation in OBD. The sample had high psychiatric vulnerability—predominantly ADHD, anxiety, and depression—exceeding epidemiological rates [20], possibly reflecting selection bias, as families seeking support for mental health problems may have been more inclined to participate. CBCL-EDP scores were elevated at baseline, aligning with previous findings on symptom complexity in at-risk youth [21–25].

Our findings demonstrate a significant reduction in CBCL-EDP scores from baseline to 12 months, supporting the potential of brief family-based programs to reduce emotional dysregulation in high-risk youth. Those clinical benefits are consistent with previous FFT research [11, 26]. Interestingly, no significant changes were observed at 6 months, which may indicate that the benefits of psychoeducation and skills practice require time to consolidate before being reflected in measurable symptom change. This pattern is consistent with interventions where sustained improvement emerges only after repeated practice and integration into family routines [27–29] While we did not collect additional indicators such as per-session attendance rates or structured satisfaction measures, the absence of dropout and sustained engagement across all sessions suggest that the intervention was feasible in a real-world CAMHS setting.

Youth with ADHD showed the greatest reductions in emotion dysregulation, which may reflect the strong overlap between ADHD-related attentional and behavioural regulation difficulties and dysregulation processes. The structured psychoeducational content, emphasis on routines, and parent–child communication strategies included in the program may have been particularly effective for this subgroup [30]. Notably, improvements were observed for emotional dysregulation even though other outcome measures, such as mood symptoms or global functioning, did not show significant change. This suggests that dysregulation may be a more proximal and sensitive marker of intervention effects, potentially preceding broader improvements in mood or overall functioning. Targeting dysregulation may therefore represent an important early intervention strategy, especially in at-risk populations where symptoms are heterogeneous and not yet syndromic.

The small sample size, non-epidemiological design, lack of control group, and significant attrition, typical in similar studies [31], limit definitive conclusions. Additionally, parent-reported psychopathology may introduce bias through symptom overreporting [32]. Multi-informant assessments would enhance reliability. Moreover, the absence of measurement on participant acceptability/satisfaction and parental practices, should be systematically assessed in future studies. Given BD’s strong heritability [21] and elevated psychopathology risk in OBD ([5, 33]–[34]), prioritizing early detection and preventive family-based interventions is essential [35]. Our study supports the feasibility and potential benefits of such family-based approaches in CAMHS, though larger controlled trials are necessary. Future research should focus on their role in disorder prevention, improving global functioning, and clarifying genetic-environmental interactions.

## Data Availability

The anonymised dataset is available from the corresponding author.
